# Effect of different positive end-expiratory pressure levels in patients undergoing laparoscopic cholecystectomy under general anesthesia

**DOI:** 10.12669/pjms.41.3.11348

**Published:** 2025-03

**Authors:** Xin Pan, Dan Wang

**Affiliations:** 1 Xin Pan Department of Anesthesiology, Affiliated Hospital of Xuzhou Medical College, Xuzhou, Jiangsu Province 221000, P.R. China; 2 Dan Wang Department of Anesthesiology, Xuzhou Hospital of Traditional Chinese Medicine, Xuzhou, Jiangsu Province 221000, P.R. China

**Keywords:** Positive end-expiratory pressure, Laparoscopic cholecystectomy, General anesthesia

## Abstract

**Objective:**

To investigate the effect of different levels of positive end-expiratory pressure (PEEP) in patients undergoing laparoscopic cholecystectomy under general anesthesia.

**Methods:**

This retrospective study included patients who underwent laparoscopic cholecystectomy under general anesthesia in the Xuzhou Hospital of Traditional Chinese Medicine from January, 2023 to March, 2024. Based on the PEEP levels, patients were grouped into 0cm group (0cmH_2_O), 5cm group (5cmH_2_O), 8cm group (8cmH_2_O), and 10cm group (10cmH_2_O). Mean arterial pressure (MAP), mean airway pressure (P_mean_), peak airway pressure (P_peak_), and blood gas status levels (oxygenation index[OI], arterial partial pressure of oxygen [PaO_2_], and arterial partial pressure of carbon dioxide [PaCO_2_]) of all four groups were measured at five minutes after the intubation (T1), five minutes after pneumoperitoneum (T2), and 30 minutes after pneumoperitoneum (T3).

**Results:**

A total of 84 patients (37 males and 47 females) were included in this study. The number of patients in the 0cm group, 5cm group, 8cm group, and 10cm group were 24, 24, 21, and 15, respectively, and there were no significant differences in the baseline data among the four groups. There were significant differences in P_mean_, P_peak_, and MAP between the four groups at T2 and T3. The increase in PEEP was accompanied by a gradual increase in P_mean_ and P_peak_ (*P*<0.05). There were significant differences in OI, PaCO_2_, and PaO_2_ among the four groups at T2 and T3. With the increase in PEEP, OI and PaO_2_ values continued to increase while PaCO_2_ continued to decrease (*P*<0.05).

**Conclusions:**

During laparoscopic cholecystectomy under general anesthesia, PEEP = 5cmH_2_O can inhibit a significant decrease in MAP while ensuring the patient’s blood gas and respiratory mechanics status, which can ensure hemodynamic stability.

## INTRODUCTION

Cholelithiasis or gallstones are one of the most prevalent disorders of the digestive system, with an incidence of 8~10%, and around 50% of cholelithiasis patients are symptomatic.^1^–^3^ Laparoscopic cholecystectomy, a commonly used clinical treatment for cholelithiasis, has the advantages of minimal trauma, high safety, mild pain, and fast postoperative recovery.^4^,^5^ However, for patients who develop cholelithiasis, invasive surgery, and general anesthesia can seriously impact organ function and cause organic damage.^6^,^7^ This can lead to functional circulatory and respiratory abnormalities, increasing the risk of perioperative complications.^6^–^8^ Therefore, implementing effective management and lung protection for patients undergoing laparoscopic cholecystectomy under general anesthesia is crucial.

Positive end-expiratory pressure (PEEP) refers to the maintenance of above atmospheric (positive) pressure at the airway opening at the end of expiration that can be set up during mechanical ventilation.^9^ Studies show that properly set PEEP in patients undergoing surgery under general anesthesia can quickly increase airway pressure, increase lung volume, avoid secondary atelectasis caused by low tidal volume ventilation, improve oxygenation status, and reduce alveolar filling-induced lung injury.^10^,^11^ Moreover, setting an appropriate level of PEEP is beneficial for achieving uniform alveolar ventilation, enhancing gas exchange capacity, reducing the consumption of pulmonary surfactant, alleviating damage to pulmonary endothelial cells, and regulating gas distribution.^12^,^13^

However, there is still no unified consensus on the optimal level of PEEP in patients who had undergone laparoscopic cholecystectomy and evidence on it is still short. This study intended to assess the effects of different levels of PEEP on respiratory mechanics and hemodynamics in patients undergoing laparoscopic cholecystectomy under general anesthesia.

## METHODS

This retrospective study included patients who underwent laparoscopic cholecystectomy under general anesthesia in Xuzhou Hospital of Traditional Chinese Medicine from January, 2023 to March, 2024. Patients were divided into the 0cm group (0cmH_2_O), 5cm group (5cmH_2_O), 8cm group (8cmH_2_O), and 10cm group (10cmH_2_O) based on different PEEP levels.

### Ethical Approval:

Ethics Committee of the hospital approved the study Ref. No. 50/2024, date: May 28, 2024).

### Inclusion criteria:

- All patients underwent laparoscopic cholecystectomy under general anesthesia.
- Complete clinical data.
- The American anesthesiologist (ASA) grading of level II-III.

### Exclusion criteria:

- Patients with concomitant renal and liver dysfunction.
- Patients with blood system and autoimmune diseases.
- Patients with malignant tumors.
- Patients with airway obstruction.
- Patients with bronchial asthma, chronic obstructive pulmonary disease, and pulmonary tuberculosis.

### Laparoscopic cholecystectomy under general anesthesia:

Patients were instructed to fast before the surgery. After entering the room, routine monitoring of electrocardiogram, body temperature, heart rate, pulse oxygen saturation, and non-invasive blood pressure was performed using an electrocardiogram monitor. The end-expiratory carbon dioxide partial pressure (PETCO2) was monitored. Local anesthesia was administered and left radial artery puncture, and catheterization were implemented, followed by monitoring of invasive blood pressure. General anesthesia was initiated by intravenous infusion of 0.2 mg/kg cisatracurium (Jiangsu Hengrui Pharmaceuticals Co., Ltd; China), 3-6 ug/kg fentanyl (Yichang Humanwell Pharmaceutical Co., Ltd; China), 0.3-0.4 mg/kg etomidate (Jiangsu Enhua Pharmaceutical Co., Ltd; China), 0.05 mg/kg midazolam (Jiangsu Enhua Pharmaceutical Co., Ltd; China). After mask oxygen ventilation for three minutes, tracheal intubation was performed, and the patient was connected to an anesthesia machine for mechanical ventilation. Remifentanil and propofol were administered intraoperatively for anesthesia maintenance, and intermittent intravenous injections of cisatracurium were done, with mean arterial pressure (MAP) maintained at baseline ± 20%. The anesthesia machine parameter setting was as follows: inhalation oxygen concentration set to 100%, flow rate 2 L/min. Respiratory parameters before carbon dioxide pneumoperitoneum: respiratory rate set to 12 beats/min, tidal volume of 8 ml/kg, respiratory ratio of 1:2. After the patient’s spontaneous breathing disappeared and under the volume-controlled ventilation (VCV) mode, tidal volume was set to 8 mL/kg, and the PEEP parameters of each group were 0cmH_2_O, 5cmH_2_O, 8cmH_2_O and 10 cmH_2_O, respectively.

***The following baseline data and surgical process-related indicators were collected from patients:***

- MAP, P_mean_, and P_peak_ levels at five minutes after the intubation (T1), five minutes after pneumoperitoneum (T2), and 30 minutes after pneumoperitoneum (T3). MAP at different time points were recorded by multi-functional monitoring device (B40I; GE Healthcare Finland Oy; USA). Levels of P_mean_, and P_peak_ at different time points were recorded using respiratory function monitoring instrument (Carestation 620; Datex-Ohmeda,Inc; USA).
- Levels of blood gas including oxygenation index (OI), arterial partial pressure of oxygen (PaO_2_), and arterial partial pressure of carbon dioxide (PaCO_2_) at T1, T2, and T3. Arterial blood samples were drawn from patients at different time points, and blood gas indicators were analyzed using blood gas analyzer (ABL90 FLEX, Radiometer Medical ApS; Denmark).

### Statistical analysis:

Data were analyzed using SPSS version 25.0 (IBM Corp, Armonk, NY, USA) and PRISM8.0 software (GraphPad, San Diego, USA). Continuous variables were reported as mean and standard deviation (SD) and count data were reported as numbers. One-way analysis of variance (ANOVA) was used to evaluate the statistical significance of continuous variable differences among the four groups, and the LSD method was used for pairwise post hoc comparison. Count data were analyzed by Chi-square test. A p-value less than 0.05 was considered statistically significant, and all reported p-values were bilateral.

## RESULTS

**Fig.1:**
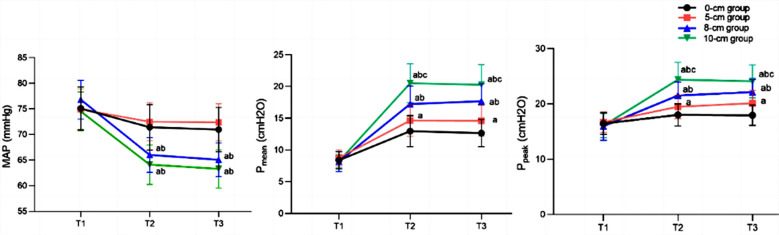
Comparison of MAP, P_mean_, and P_peak_ levels at three different time points; Compared with the 0cm group, ^a^P<0.05; compared with the 5cm group, ^b^P<0.05; compared with the 8cm group, ^c^P<0.05; MAP: mean arterial pressure; P_mean_: mean airway pressure; P_peak_: peak airway pressure.

**Fig.2:**
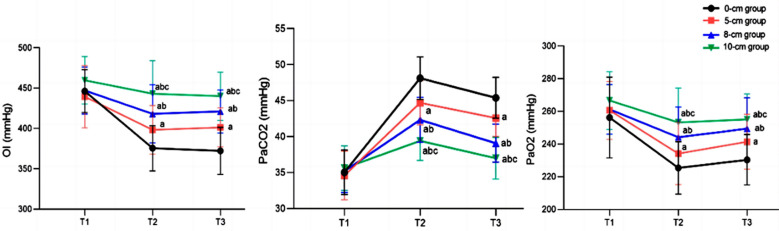
Comparison of blood gas status at three different time points; Compared with the 0cm group, ^a^P<0.05; compared with the 5cm group, ^b^P<0.05; compared with the 8cm group, ^c^P<0.05; OI: Oxygenation index; PaCO_2_: arterial partial pressure of carbon dioxide; PaO_2_: arterial partial pressure of oxygen.

Clinical records of 84 patients (37 males and 47 females) were included in this study. The age of the patients ranged from 22 to 77 years, with a mean of 50.07 ± 13.37 years. There were 24 cases in the 0cm group, 24 cases in the 5cm group, 21 cases in the 8cm group, and 15 cases in the 10cm group, with no significant difference in baseline data among the four groups (*P*>0.05), Table-I. There was no significant difference in MAP, P_mean_, and P_peak_ levels among the four groups at T1 (*P*>0.05). P_mean_, P_peak_, and MAP at T2 and T3 differed significantly between the groups, and the increase in PEEP was associated with the increase in P_mean_ and P_peak_ (*P*<0.05). MAP of patients in the 0cm group and the 5cm group was higher than that of 8cm group and 10cm group (*P*<0.05), Fig.1. There was no significant difference in OI, PaCO_2_, and PaO_2_ among the four groups at T1 (*P*>0.05). However, these parameters became significantly different between the groups at T2 and T3, and as PEEP increased, OI and PaO_2_ continued to increase, while PaCO_2_ continued to decrease (*P*<0.05), Fig.2.

**Table-I:** Comparison of demographic characteristics for four groups.

Characteristics	0cm group (n=24)	5cm group (n=24)	8cm group (n=21)	10cm group (n=15)	F/χ^2^	P
Male (yes)	7 (29.17)	13 (54.17)	8 (38.10)	9 (60.00)	5.004	0.172
Age (year)	53.25±12.52	47.92±12.86	47.95±14.46	51.40±14.09	0.881	0.455
BMI (kg/m^2^)	24.55±4.59	24.87±4.43	23.46±3.40	25.42±3.52	0.764	0.517
***ASA classification (n)***						
II	21 (87.50)	20 (83.33)	19 (90.48)	14 (93.33)	1.033	0.793
III	3 (12.50)	4 (16.67)	2 (9.52)	1 (6.67)		
Smoking history (yes)	3 (12.50)	4 (16.67)	2 (9.52)	5 (33.33)	4.071	0.254

## DISCUSSION

Study has compared two different levels of PEEP (5cm vs 10cm H_2_O PEEP) in patients undergoing laparoscopic cholecystectomy and found that 10cm H_2_O PEEP improves oxygenation during surgery, without significant hemodynamic changes.^14^ Different from Sen’s study, we also studied the effect of 0cm and 8cm H_2_O PEEP. In this study, it was shown that in these patients, an increase in PEEP was associated with a gradual increase in P_mean_, P_peak_, OI, and PaO_2_, while PaCO_2_ continued to decrease. However, the MAP of patients in the 0cm and 5cm groups was markedly higher than that of patients in the 8cm and 10cm groups. Our results confirm that different levels of PEEP can affect the hemodynamic and respiratory status of patients undergoing laparoscopic cholecystectomy under general anesthesia. Our results demonstrated that PEEP of 5cmH_2_O can efficiently minimize the decrease in MAP while ensuring good blood gas and respiratory mechanics status of the patient, which has a positive significance for maintaining hemodynamic stability.

Arora V et al.^13^ used 6cmH_2_O PEEP in patients undergoing laparoscopic cholecystectomy and showed this intervention did not result in a significant difference in hemodynamic parameters, peak airway pressure, and dynamic compliance compared to patients who were not on PEEP. These results implied that PEEP does not affect the hemodynamic status and gas exchange function of patients, which is somewhat different from the results of our study. This discrepancy may be related to differences in sample size and research design. Arinalp HM et al.^15^ showed that PEEP of 8cmH_2_O was linked to minimal postoperative lung function damage in patients undergoing laparoscopic cholecystectomy. However, our study showed that the blood gas state and P_mean_ and P_peak_ were significantly better in patients who received PEEP of 10cmH_2_O. Previous studies demonstrated that high levels of PEEP are less damaging to lung function.^15^-^17^ In addition, Kemerci PU et al.^18^ also confirmed that a PEEP of 10cmH_2_O is beneficial for improving blood gas status and heart rate levels, which is consistent with our results. Yilmazlar et al.^19^ also found that a PEEP level of 10cmH_2_O can more effectively improve the respiratory mechanics of patients undergoing laparoscopic cholecystectomy.

However, our study shows that a PEEP of 5cmH_2_O is also effective in preventing significant fluctuations in MAP. We may speculate that while PEEP can improve lung ventilation and oxygenation function if it is too high, it can lead to a significant increase in intrathoracic pressure, affecting venous return, reducing the amount of return heart blood, and ultimately causing a decrease in cardiac output.^20^,^21^ Moreover, excessive PEEP can also damage airway pressure and increase the risk of complications such as pneumothorax.^22^,^23^ Therefore, based on our results, the PEEP setting of 5cmH_2_O is the most safe and efficient during the surgery.

However, Ciftci B et al.^24^ explored the impact of different levels of PEEP on respiratory parameters-related indicators in patients undergoing laparoscopic cholecystectomy and showed that in patients on PEEP of 5 cmH_2_O or 8cmH_2_O, levels of lung function-related indicators at one hour, six hours, and 24 hours after the surgery were lower, compared to patients who were not on PEEP. These results imply that PEEP has a negative impact on postoperative lung function in patients undergoing elective laparoscopic cholecystectomy. Such discrepancies in results suggest that a more tailored approach needs to be adopted in surgical patients who require PEEP. Indeed, a meta-analysis by Li X et al.^25^ suggested that individualized PEEP and recruitment procedures are much more effective low tidal volume ventilation strategies compared to low PEEP without recruitment procedures. Although maintaining a certain amount of PEEP seems crucial, selecting and fine-tuning optimal PEEP levels remains challenging.

From all above, the current study provides more evidence for further research on the clinical application of PEEP in patients undergoing laparoscopic cholecystectomy under general anesthesia, and helps clinicians make appropriate treatment decisions.

### Limitations:

Firstly, it is a small-sample size single-center retrospective study. Therefore, possible selection bias may have occurred. Secondly, the impact of different PEEP levels on postoperative lung function and complications in patients was not analyzed. Thirdly, respiratory management during the consultation period and immediately after the surgery may have been uncoordinated. The results of this study are only applicable to intraoperative ventilation management. Finally, the impact of different PEEP levels on the long-term functional recovery of patients was not analyzed

## CONCLUSION

During laparoscopic cholecystectomy under general anesthesia, PEEP = 5cmH_2_O can inhibit a significant decrease in MAP while ensuring blood gas and respiratory mechanics status, which can ensure the overall hemodynamic stability of patients.

### Recommendations:

Therefore, prospective multi-center studies are still needed to validate the conclusions of this study and to further explore the optimal level of PEEP.
